# Breast milk–saliva interactions in shaping early mucosal immunity

**DOI:** 10.3389/fimmu.2025.1679865

**Published:** 2026-01-12

**Authors:** Seon Bin Song, Jae Young Kim

**Affiliations:** Department of Life Science, Gachon University, Seongnam, Kyeonggi-Do, Republic of Korea

**Keywords:** breast milk, neonatal saliva, human milk oligosaccharides, mucosal immunity, xanthine oxidase, hypoxanthine, microbiota, glycosylation

## Abstract

The neonatal period is characterized by immunological immaturity, rendering newborns vulnerable to infections at mucosal surfaces such as the oral and gastrointestinal tracts. Breast milk, traditionally recognized for its nutritional and immunological roles, and neonatal saliva, once considered a passive secretion, interact biochemically to create a synergistic defense system. These interactions include the xanthine oxidase–lactoperoxidase system, which generates hydrogen peroxide and hypothiocyanite, compounds with potent antimicrobial and immunomodulatory properties. Beyond immediate antimicrobial action, these factors regulate mucosal barrier integrity, immune gene expression, and glycosylation processes. This review integrates recent findings on the compositional elements, biochemical pathways, and immunological outcomes of breast milk–saliva interactions, emphasizing their roles in shaping early mucosal immunity, establishing beneficial microbiota, and promoting immune tolerance. These insights also highlight potential strategies to enhance artificial infant formulas and therapeutic interventions for neonatal care.

## 1 Introduction

The neonatal period represents a critical window for immune system development, yet is marked by structural and functional immaturity of both innate and adaptive immunity. Mucosal surfaces, especially the oral and gastrointestinal tracts, are the primary entry points for pathogens, rendering newborns highly susceptible to infection (1). While the immunological benefits of breast milk are well established (2), and neonatal saliva is increasingly recognized as bioactive, their biochemical and immunological interaction has received limited attention (1, 3, 4).

We hypothesize that the dynamic interplay between breast milk and neonatal saliva forms an evolutionarily optimized mucosal defense system that actively modulates innate immunity, microbial colonization, and epithelial barrier function. This review integrates emerging evidence to support this concept, outlining the biochemical pathways and immunological consequences of breast milk–saliva interactions, and exploring their potential clinical applications.

## 2 Characteristics of the neonatal immune system

The transition from the sterile intrauterine environment to the microbe-rich external world represents a profound immunological challenge for neonates. Rather than mounting robust inflammatory responses similar to adults, the neonatal immune system adopts a tolerance-oriented strategy, which is an adaptation shaped by developmental constraints and evolutionary priorities. Neonatal epithelial barriers are immature, with wider intercellular junctions, reduced mucus secretion, and lower levels of antimicrobial peptides, rendering mucosal surfaces highly permeable to pathogens (4, 5). Phagocytic functions, including chemotaxis and oxidative burst, are diminished, and Toll-like receptor (TLR) signaling pathways remain underdeveloped, leading to insufficient cytokine production and poor pathogen clearance (6, 7). Humoral and cellular components of adaptive immunity also show marked immaturity. Neonates produce minimal endogenous antibodies, with delayed class switching. Protection is largely conferred by transplacental IgG and secretory IgA (sIgA) derived from breast milk (3, 8). T cells exhibit a Th2-skewed profile, minimizing inflammation, while increased frequencies of regulatory T cells (Tregs) promote tolerance during initial microbial colonization (6). Though protective, this profile compromises efficient responses to pathogens.

Why do neonates suppress inflammatory responses at such a vulnerable time? The answer lies in the physiology of the neonate. Fragile tissues, immature vasculature, and underdeveloped inflammatory resolution pathways make uncontrolled immune activation potentially lethal, which risks mucosal injury, systemic cytokine storms, and sepsis (9, 10). Tolerance, rather than aggression, emerges as a safer survival strategy. This strategy also facilitates the establishment of beneficial microbes such as *Bifidobacterium* and *Lactobacillus*. Failure to properly seed these commensals early in life increases susceptibility to allergic, autoimmune, and metabolic diseases later in life (11–13).

The immunological conservatism of the neonate is also shaped, in part, by its metabolic profile. Rapid growth and organogenesis dominate energy expenditure in early life, limiting available resources for immune defense (14). Even during infection, energy is preferentially allocated to tissue maintenance rather than immunity. Furthermore, neonates are born with minimal vitamin A stores, which is an essential cofactor for epithelial differentiation and tolerance induction via CD103^+^ dendritic cells (15, 16). Interestingly, the neonatal gut harbors a higher abundance of group 2 innate lymphoid cells (ILC2s), which support tissue repair but also initiate allergic responses. While this ILC2 dominance may promote homeostasis, it may simultaneously predispose neonates to allergic sensitization (17, 18).

Taken together, the neonatal immune system is not merely immature but is strategically adapted to promote tolerance, accommodate microbial colonization, and conserve energy. Rather than a deficit, this configuration represents an evolutionarily optimized immune profile for surviving the first critical months of life (19). Understanding this distinct immunological landscape is essential for evaluating how exogenous factors, such as breast milk and neonatal saliva, interface with and shape early-life mucosal immunity.

## 3 Immunological components of human milk

Human milk is not merely a nutritional fluid but a sophisticated immunological medium, finely tuned to support neonatal development and mucosal defense. Its composition reflects an evolutionary strategy to provide immune protection, guide microbial colonization, and support tissue maturation during a period of immunological vulnerability. Key bioactive constituents, including immunoglobulins, cytokines, growth factors, glycans, and immune cells, interact synergistically to establish mucosal tolerance and antimicrobial readiness.

### 3.1 Immunoglobulins: passive protection and immune education

Among milk-derived immune components, secretory IgA (sIgA) plays a central role in mucosal immunity, as shown by human observational studies. It binds to pathogens and toxins without triggering inflammation, thereby maintaining barrier integrity and facilitating immune exclusion. This mechanism has been characterized primarily in *in vitro* experiments (3). Human measurements indicate that colostrum contains especially high levels of sIgA (about 10 g/L), which decline over time. Strikingly, mothers in environments with higher microbial exposure, such as low- and middle-income countries, produce more diverse and abundant milk-borne IgA, supporting the “enteromammary link” hypothesis -whereby maternal mucosal exposures shape the antibody repertoire passed to the infant (20).

IgG and IgM are also present in human milk at lower concentrations. Their presence is well documented in human samples (3), whereas evidence for opsonization and activation of complement comes predominantly from preclinical work, including *in vitro* and animal studies (21). Features of neonatal gastrointestinal physiology, including relatively low acidity and protease activity, suggest that these antibodies can remain functionally intact (22); this interpretation is supported mechanistically mainly by *in vitro* data. In addition, protective proteins such as alpha 1 antitrypsin and trypsin inhibitors have been shown in preclinical studies to shield immune factors from degradation, thereby enhancing their delivery to the gut mucosa (23).

### 3.2 Cytokines, growth factors, and antimicrobial proteins

Milk-borne cytokines help balance tolerance and immunity. Preclinical studies indicate that anti-inflammatory mediators such as IL-10 and TGF-β facilitate the development of oral tolerance and promote the expansion of regulatory T cells (Tregs), while controlled levels of pro-inflammatory cytokines (e.g., IL-6, TNF-α) enhance antimicrobial defense without inducing tissue injury (24, 25). Human measurements show that growth factors present in milk, such as EGF, IGF, erythropoietin, and G-CSF, are detectable and abundant. Observational human data, together with supporting preclinical work, indicate that these factors are involved in intestinal development, maintenance of barrier integrity, and epithelial repair. For instance, EGF concentrations in colostrum are orders of magnitude higher than maternal serum levels, and this difference is associated in human studies with intestinal maturation and improved nutrient absorption, with mechanisms characterized mainly in preclinical systems (26). In terms of mechanism, EGF binds to EGFR on intestinal epithelial cells and activates canonical MAPK/ERK and PI3K/AKT signaling cascades that support cell-cycle entry, restitution, and survival; these pathways have been characterized primarily in preclinical systems and summarized in representative reviews (27). Reports from cell and animal models further indicate that EGF can enhance expression and assembly of tight-junction constituents (e.g., occluding, claudins), promote epithelial migration and cytoskeletal remodeling, and increase mucus production, thereby contributing to barrier restoration after injury (28–30). Human analyses confirm that innate immune proteins such as lactoferrin, lysozyme, and defensins are present in milk. Their antimicrobial mechanisms, including iron chelation and membrane disruption for lactoferrin, peptidoglycan targeting for lysozyme, and broad-spectrum activity for defensins, have been demonstrated primarily *in vitro* and in animal models, while human observational studies align with a protective role during lactation (31, 32).

### 3.3 Human milk oligosaccharides: multifunctional glycans

Human milk oligosaccharides (HMOs) are the third most abundant solid component in human milk and are recognized as immunologically active glycans rather than inert nutrients (33, 34). Over 200 structurally distinct HMOs have been identified (33, 34). *In vitro* studies demonstrate that specific HMOs function as pathogen decoys, preventing adherence of microbes to epithelial cells, for example, 2′-FL inhibiting E. coli and Campylobacter binding (33). Both *in vitro* and animal model data indicate that HMOs selectively support beneficial microbes such as Bifidobacterium and Lactobacillus and promote short-chain fatty acid (SCFA) production, which is associated with Treg induction and epithelial health (35). At the immune-cell level, *in vitro* experiments show that 2′-FL can downregulate TNF-α and IL-6 in macrophages via TLR4 signaling, and that 3′-SL can modulate dendritic cells and T-cell polarization (36, 37). Maternal FUT2 genotype (secretor status) is a major determinant of fucosylated HMO profiles, which could alter microbial selectivity and downstream interactions at the milk–saliva interface. Maternal secretor status determined by FUT2 genotype is a major source of interindividual variation in human milk oligosaccharides. Secretor positive mothers produce higher levels of 2′-fucosyllactose and related fucosylated structures, whereas non-secretor mothers have markedly lower concentrations of these glycans (33, 34, 38). Human cohort studies further associate maternal secretor status with infant gut community structure, including enrichment of infant-adapted bifidobacteria in secretor dyads (39, 40). Given that fucosylated HMOs act as pathogen decoy ligands and selective substrates for bifidobacteria, this genetic modulation likely conditions how milk glycans interface with salivary substrates and oral–gut colonization during breastfeeding (33, 34). Although direct tests at the milk–saliva interface remain limited, these observations suggest a plausible route by which maternal genotype can influence the magnitude and specificity of milk–saliva interactions. Beyond HMOs, *in vitro* work suggests that glycoproteins such as lactadherin facilitate clearance of apoptotic cells, and mucins (e.g., MUC1, MUC4) prevent microbial adhesion at mucosal surfaces. Finally, human cohort and observational studies indicate that these glycan-rich molecules are dynamically shaped by maternal health and environmental exposures, thereby contributing to a personalized layer of immune protection (41).

Although neonatal mucins are present, human comparative analyses indicate lower levels of sialylation and fucosylation relative to adults, which may limit antimicrobial and immunomodulatory capacity. HMOs, particularly 2′-fucosyllactose and sialylated structures, have been shown in preclinical and *in vitro* systems to act as decoy receptors that block pathogen adhesion and to modulate immune signaling (33, 42). In animal models, 2’-FL has been shown to inhibit Campylobacter jejuni binding while increasing MUC2 expression and goblet cell numbers, indicating its role in mucosal barrier maturation (43). In parallel, milk-derived cytokines such as TGF-β and EGF have been reported to influence glycosyltransferase expression (e.g., FUT2, ST3Gal, ST6GalNAc) and may facilitate endogenous remodeling of mucin glycans in the neonatal gut (44, 45). Collectively, these findings support the view that breast milk functions not only as a glycan source but also as a developmental cue shaping neonatal mucosal glycosylation, while much of the mechanistic evidence derives from preclinical studies.

Because concentrations of sIgA, growth factors, and HMOs shift across lactation, the magnitude and selectivity of milk–saliva interactions are likely to vary over time. Across early lactation, the composition of human milk changes in ways that are relevant to its interaction with neonatal saliva. Concentrations of secretory IgA are highest in colostrum and decline in mature milk, and growth factors such as EGF and IGF also decrease over time, which may modulate the intensity of mucosal signaling during the early postnatal period (26, 46–48). Human milk oligosaccharides display stage specific patterns and structural diversity, with quantitative shifts across colostrum, transitional, and mature milk that could alter microbial selectivity and decoy functions as lactation progresses (33, 34). Cytokines, including TGF-β and IL-10, are detectable throughout lactation, though reported levels vary with stage and maternal factors, suggesting time dependent effects on tolerogenic programming (24, 25, 41).

### 3.4 Immune cells: cellular mediators of tolerance and defense

Beyond soluble components, human milk contains viable immune cells, particularly in colostrum, as shown in human analyses. Macrophages constitute the predominant leukocyte subset in these samples. Human analyses confirm the presence of viable leukocytes in colostrum; however, evidence for their post ingestion activity and transepithelial passage in human infants remains indirect. Claims that milk leukocytes remain active after ingestion and traverse the neonatal gut epithelium during early postnatal life are based mainly on preclinical or *ex vivo* observations (49, 50). In cell based and other preclinical studies, milk-derived macrophages exhibit antigen-presenting functions, produce anti-inflammatory cytokines such as IL-10 and TGF-β, and can kill pathogens including *Staphylococcus aureus* (49, 50). Under IL-4 stimulation *in vitro*, these cells can acquire dendritic-like features consistent with a potential role in mucosal immune education. Human milk also contains extracellular vesicles, including exosomes carrying microRNAs, cytokines, and growth factors; their presence is well documented in human samples, while immunoregulatory effects are inferred largely from preclinical work (51). Taken together, available evidence supports the view that human milk provides more than passive immunity. It functions as an active and developmentally responsive immunological interface between mother and infant. Its molecular and cellular components appear to work in concert to balance tolerance, encourage microbial symbiosis, and prepare the neonatal mucosa for extrauterine life, and these features provide a foundation for understanding how milk–saliva interactions may further enhance or modulate these protective effects.

## 4 Immunological components of neonatal saliva

Once regarded as a passive digestive fluid, neonatal saliva is now recognized as an immunologically active secretion that participates in shaping early mucosal defense (52). Despite being compositionally distinct from adult saliva, it harbors a set of bioactive molecules, including mucins, innate immune proteins, nucleotide precursors, and microbiota-modulating metabolites that interact not only with mucosal tissues but also synergize with breast milk to reinforce host protection (4, 53).

### 4.1 Mucin and glycan signatures: the nascent glycocalyx

Neonatal saliva contains mucins, primarily MUC5B and MUC7, which lubricate mucosal surfaces and block pathogen adherence through densely glycosylated O-linked chains (54, 55). Sialylated and fucosylated mucin residues have been shown *in vitro* to act as decoy receptors for a wide range of pathogens, including *Helicobacter pylori*, *Campylobacter jejuni*, influenza viruses, and enterotoxigenic *E. coli* (56, 57). Compared with adults, neonatal mucins, particularly in MUC7, exhibit lower sialylation and fucosylation in human samples (58), a difference that may diminish barrier function, although direct *in vivo* confirmation is limited. This under-glycosylation likely reflects developmental immaturity and may create a window in which maternal glycans (e.g., from breast milk) can functionally complement the neonatal mucosal surface. In addition, interactions of sialylated mucins with Siglecs and of fucosylated glycans with lectin receptors such as DC-SIGN and MGL are predominantly supported by *in vitro* or preclinical studies, suggesting potential roles in immune tolerance, antigen sampling, and immune calibration (59, 60).

### 4.2 Immunoglobulins and antimicrobial molecules

Neonatal saliva contains innate immune components such as lysozyme, lactoperoxidase (LPO), and defensins, although at lower levels than in adults, as shown in human measurements. In the presence of hydrogen peroxide and thiocyanate (SCN^−^), LPO generates hypothiocyanite (OSCN^−^), a well-characterized antimicrobial product established in preclinical systems. Although neonatal saliva alone may not fully activate this pathway, *in vitro* studies indicate that mixing with breast milk, which provides xanthine oxidase (XO) and purine substrates, can rapidly produce OSCN^−^ at mucosal interfaces (4, 61). Consistent with this, secretory IgA detected in neonatal saliva is largely attributed to transfer from breast milk and supports immune exclusion without provoking inflammation (62, 63). Taken together, these observations suggest that breastfeeding can amplify the antimicrobial functions detectable in neonatal saliva, while acknowledging that much of the mechanistic evidence derives from preclinical studies.

### 4.3 Nucleotide precursors: immune modulators and microbial substrates

A defining biochemical feature of neonatal saliva is the markedly elevated level of purine metabolites, particularly hypoxanthine and xanthine, which have been reported at more than tenfold the concentrations found in adults based on human measurements (4). This elevation likely reflects rapid postnatal nucleic acid turnover together with relatively low expression of xanthine oxidoreductase (XOR) in neonatal oral tissues, leading to metabolite accumulation; this interpretation is inferred from limited expression data. Preclinical studies indicate that extracellular conversion of hypoxanthine to adenosine can modulate macrophage function through A2A receptor signaling, increasing IL-10 and reducing TNF-α, and that purine derivatives and related nucleotide precursors can fuel the growth of beneficial taxa such as Bifidobacterium and Lactobacillus (64, 65). Taken together, these observations suggest that neonatal salivary purines may contribute both to immune regulation and to microbial nutrition, potentially shaping a mucosal milieu conducive to tolerance and the establishment of beneficial microbiota, while acknowledging that much of the mechanistic evidence comes from preclinical work.

Gestational age has been associated with differences in salivary mucin glycosylation and purine metabolites, features that may shift pathway flux during early feeding. Gestational age is associated with differences in neonatal salivary biochemistry and oral microbial ecology, which could in turn modulate milk–saliva reactions. Compared with term infants, preterm populations show distinct salivary proteomic and metabolomic profiles and altered early oral microbiota, features that may influence substrate availability for biochemical pathways and microbial adhesion dynamics (62, 66, 67). For example, neonates exhibit elevated salivary purines such as hypoxanthine and xanthine relative to adults, and these metabolites participate in the xanthine oxidase–LPO pathway *in vitro*; whether their postnatal trajectories differ by gestational age and meaningfully alter pathway flux requires prospective study (4). Taken together, maternal FUT2 genotype and gestational age represent complementary axes of variability that likely shape both the microbial selectivity of milk glycans and the biochemical context provided by neonatal saliva.

### 4.4 Environmental modulators of salivary immunity

The immunological composition of neonatal saliva is not fixed; rather, it is highly responsive to environmental influences such as birth mode, feeding type, and perinatal antibiotic exposure. Vaginal delivery exposes neonates to maternal vaginal and gut microbiota, facilitating colonization by genera like *Streptococcus*, *Lactobacillus*, and *Bifidobacterium* (66). Cesarean delivery shifts the microbial profile toward skin-associated taxa such as *Staphylococcus*, with downstream effects on salivary immunity (67). Breastfeeding enhances the colonization of beneficial oral microbiota by supplying oligosaccharides, immunoglobulins, and antimicrobial proteins. In contrast, formula-fed infants exhibit reduced microbial diversity and resilience (68, 69). Additionally, intrapartum antibiotic administration alters microbial succession, decreasing colonization by beneficial taxa and increasing antibiotic-resistant *Proteobacteria* (70, 71). These findings reinforce the notion that salivary immunity is plastic and maternally modulated, and that breast milk plays a central role in shaping its functional composition.

Feeding modality also conditions salivary substrates and oral ecology, which in turn may modulate the effective output of milk–saliva pathways. Neonatal saliva also shows features that may change the effective output of milk–saliva reactions. Relative to adults, neonatal saliva contains elevated purine metabolites such as hypoxanthine and xanthine, as well as lower overall glycosylation of mucins, which together can influence the activation of the lactoperoxidase pathway and the quality of mucosal barriers; limited data indicate that these features evolve with postnatal age (4, 58, 62). Feeding modality is associated with differences in the infant oral microbiota, with breastfeeding linked to enrichment of streptococci and lactobacilli and formula feeding associated with distinct community structures; these ecological differences could condition how salivary substrates and milk bioactives interact at the oral interface (66–69).

Cesarean-associated shifts in early oral communities, together with perinatal antibiotic exposure, may attenuate elements of milk-mediated protection by reducing the abundance of infant-adapted taxa or altering sensitivity to oxidant-mediated defenses. Delivery mode and antibiotic exposure are important modifiers of early mucosal ecology and may shape the functional output of milk–saliva interactions. Human observational studies report that cesarean delivery is associated with distinct early oral and gut microbiota, with reduced exposure to maternal vaginal and intestinal taxa and a relative enrichment of skin associated organisms; several cohorts also describe delayed microbial maturation and altered metabolite profiles (66, 67, 72, 73). In parallel, intrapartum or early life antibiotic exposure has been linked to decreased colonization by commensal streptococci and bifidobacteria and to expansion of proteobacterial taxa, including strains that carry resistance determinants (70, 71).

These ecological differences could modify how human milk oligosaccharides, immunoglobulins, lactoferrin, and other milk constituents interface with neonatal saliva at the oral surface. For example, a community with fewer infant adapted bifidobacteria may exhibit reduced capacity to use fucosylated and sialylated glycans, and a shift toward proteobacteria may alter sensitivity to oxidants generated by the LPO system in preclinical models (4, 33, 61). Although direct tests at the milk–saliva interface in human infants are limited, these observations suggest that cesarean associated dysbiosis and early antibiotic exposure could attenuate some elements of milk mediated protection, whereas direct breastfeeding might help restore ecological and biochemical balance through repeated delivery of milk bioactives and viable milk associated microbes (74, 75). Prospective studies that integrate delivery mode, antibiotic exposure, feeding practices, and longitudinal oral sampling are needed to determine whether these factors act synergistically or antagonistically with milk–saliva pathways and to identify modifiable targets for clinical intervention.

Neonatal saliva is compositionally immature yet retains immunological capacity best understood in concert with breast milk. Considered together, these fluids function as a coordinated system that protects mucosa, shapes early microbial colonization, and influences immune signaling. Key salivary constituents and their variability are summarized in **Table 1**.

**Table 1:** Bioactive components of neonatal saliva and their immunological roles.

Component	Major source	Function	Immunological or microbial effect
Mucin (MUC5B, MUC7)	Oral epithelium	Barrier protection, pathogen trapping	↓ in glycosylation → impaired pathogen binding
Hypoxanthine, Xanthine	Endogenous metabolism	Purine salvage, substrate for XO	Anti-inflammatory (via A2A); H_2_O_2_/OSCN^−^ generation
sIgA	Maternal breast milk	Pathogen neutralization, immune exclusion	Maintains mucosal homeostasis
Lysozyme, LPO	Salivary glands	Antibacterial enzyme systems	Synergize with H_2_O_2_ to produce OSCN^−^
Nucleotides, Nucleosides	Endogenous + milk	Microbial growth substrates	↑ growth of *Bifidobacterium*, ↓ pathogens
Microbiota-derived metabolites	Commensals	Immune modulation	SCFA production, tolerance induction

Compiled and adapted from key studies cited in the main text, including Al-Shehri et al. (4)Bobek & Situ (55), and Sweeney et al. (61).

## 5 Biochemical and immunological synergy between breast milk and neonatal saliva

**Figure 1:**
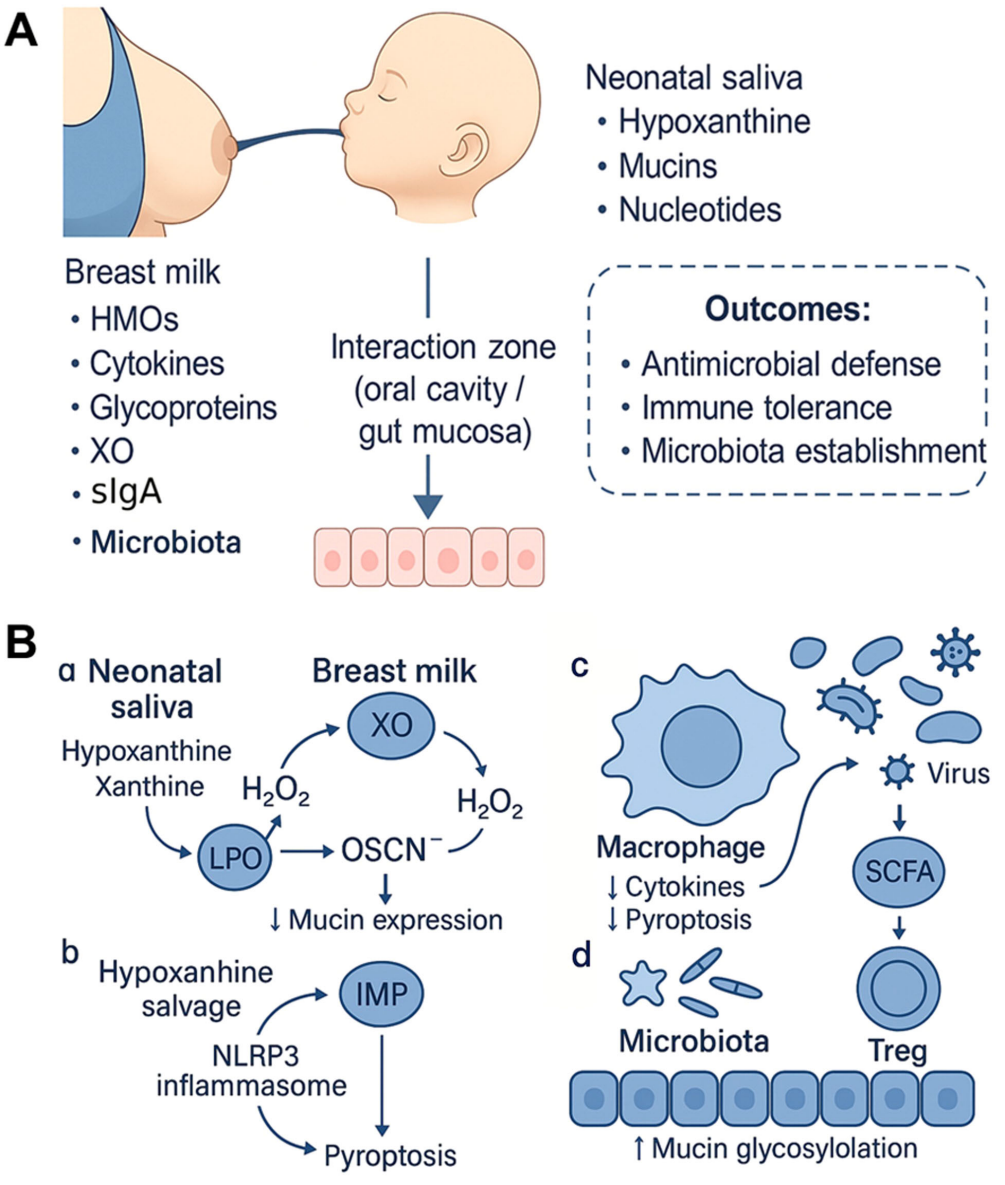
Overview and mechanistic model of breast milk–saliva synergy in early mucosal immunity. **(A)** Systems overview. Breast milk delivers HMOs, cytokines, glycoproteins, XO, and immunoglobulins (sIgA, sIgM, sIgG), with sIgA as the predominant class in human milk. Neonatal saliva contributes hypoxanthine, mucins, and nucleotides. These inputs meet at the oral cavity and upper gut mucosa to support three coordinated outcomes: antimicrobial defense, immune tolerance, and establishment of a beneficial microbiota. **(B)** The cellular and molecular mechanisms. (a) XO–LPO–OSCN^−^ axis: Breast-milk XO uses salivary purines to generate H_2_O_2_, which fuels LPO to produce OSCN^−^; downstream effects include antimicrobial activity and modulation of mucin expression. (b) Hypoxanthine salvage pathway: Under inflammatory cues, hypoxanthine is salvaged to IMP, a shift linked to reduced NLRP3 inflammasome activation and attenuation of pyroptosis. (c) Immune modulation: Microbial products and redox signaling influence macrophage responses, lowering pro-inflammatory cytokines and pyroptosis; SCFAs are shown as an exemplar output of microbiota metabolism. (d) Microbiota–mucosa interface: HMOs favor beneficial taxa and SCFA production that support Treg induction; epithelial outcomes include increased mucin glycosylation and barrier support.

The neonatal period represents a critical window during which immune tolerance must be carefully balanced against the need for pathogen defense. At this vulnerable stage, the infant’s mucosal surfaces are simultaneously colonized by microbes, exposed to environmental antigens, and reliant on maternally derived inputs for protection. Rather than operating independently, breast milk and neonatal saliva act in biochemical and immunological concert, forming an evolutionarily refined system that enhances early mucosal immunity, promotes microbial symbiosis, and may contribute to mucosal protection. These interactions are temporally dynamic; their strength and selectivity vary with lactation stage, maternal and infant context, and feeding modality, while acknowledging that direct longitudinal evidence linking temporal composition to functional outcomes remains limited and warrants prospective study (74, 75). An overview of the milk–saliva interactions is shown in **Figure 1A**, and the cellular and molecular mechanisms are detailed in **Figure 1B**.

### 5.1 Cooperative antimicrobial network supporting intestinal microbial colonization: the XO-purine-LPO axis

One prominent example of this synergy is the XO-purine derivative-LPO system. Human measurements indicate that neonatal saliva contains high hypoxanthine and xanthine, whereas breast milk provides XO. *In vitro* mixing of these fluids rapidly generates hydrogen peroxide (H_2_O_2_), which then fuels the LPO-SCN^−^ reaction to produce hypothiocyanite (OSCN^−^), a well-characterized antimicrobial oxidant (4, 76). In preclinical models, this pathway yields a localized antimicrobial effect against diverse bacteria, fungi, and viruses, and H_2_O_2_/OSCN^−^ have also been shown to influence epithelial integrity, redox balance, and mucin gene expression (77, 78). Collectively, these findings support the concept that biochemical crosstalk between breast milk and neonatal saliva can generate multipurpose effectors that couple microbial control with tissue conditioning, while much of the mechanistic evidence still derives from preclinical studies.

### 5.2 Hypoxanthine as an immune modulator

Beyond serving as a substrate for antimicrobial generation, hypoxanthine has been shown in preclinical studies to regulate macrophage responses. Under inflammatory stimulation, hypoxanthine can be salvaged to inosine monophosphate (IMP), a shift associated with reduced *de novo* purine synthesis, enhanced oxidative phosphorylation, and repression of NLRP3 inflammasome activation *in vitro* (79, 80). This metabolic redirection has been linked to preserved viability and motility and to attenuation of pyroptosis and excessive cytokine release in preclinical models. When nitric oxide suppresses XOR activity, the salvage pathway appears preferentially engaged, suggesting a connection between purine metabolism and anti-inflammatory polarization in neonatal macrophages (81). Taken together, these data raise the possibility that hypoxanthine-rich neonatal saliva, especially when oxidation to uric acid is limited by low XOR expression, may bias mucosal immune responses toward resolution rather than escalation.

### 5.3 Salivary feedback and bidirectional modulation of breast milk

Emerging evidence suggests that the interaction between neonatal saliva and breast milk may be bidirectional. Purine derivatives and microbial products in neonatal saliva can influence mammary epithelial cells, potentially modulating the composition of breast milk during active breastfeeding (4). This dynamic feedback loop is supported by observed shifts in the breast milk metabolome, including metabolites involved in vitamin E, B12, and tryptophan metabolism (82), all of which play roles in immune development, antioxidant defense, and neurodevelopment. Additionally, microbial fermentation of HMOs by infant-type bifidobacteria (e.g., *B. infantis, B. longum, B. bifidum*) can release sialic acid and fucose. Although direct evidence of their incorporation into mucin biosynthesis remains limited, it is plausible that these monosaccharides are recycled into the mucosal environment and may contribute to mucin production or glycosylation under specific environmental conditions (83–85).

### 5.4 Breast milk microbiota and early colonization: human evidence and mechanistic context

Human observational studies report viable and DNA detectable bacteria in fresh human milk, including bifidobacteria, streptococci, staphylococci, and lactobacilli, with overlap at the strain or species level in infant oral and gut samples that is consistent with maternal to infant transfer (74, 75, 86–88). Proposed routes include retrograde flow during breastfeeding, cellular carriers, and an entero mammary linkage in which maternal mucosal exposures influence milk composition; because milk is a low biomass sample, strict negative controls and strain resolved approaches are required to minimize contamination risk (75, 86, 88).

HMOs select for infant adapted bifidobacteria that harbor glycan utilization loci, a conclusion supported mechanistically by preclinical and *in vitro* work and synthesized in human focused reviews (33, 89, 90). These microbes generate short chain fatty acids and lactate that are linked in preclinical models to epithelial health and regulatory T cell induction (90). Observational studies associate breastfeeding with microbial communities enriched for beneficial taxa and with reduced risk of gastrointestinal and respiratory infection, while direct attribution to specific consortia awaits interventional trials (33, 74, 75). Methodological variables such as delivery mode, intrapartum antibiotics, maternal diet, and lactation stage should be captured prospectively to reduce confounding and to harmonize analyses across cohorts (75, 86, 88).

### 5.5 Crosstalk between breast milk microbiota and neonatal saliva

Breastfeeding brings milk associated microbes into immediate contact with neonatal saliva and oral mucosa. *In vitro* and *ex vivo* studies indicate that salivary pH, ions, and enzymes influence bacterial adhesion and biofilm behavior, while milk components such as secretory IgA, lactoferrin, and mucin like glycoproteins modulate aggregation and immune recognition (33, 90). Neonatal saliva contains elevated purine metabolites and nucleic acid precursors that can serve as salvage substrates for microbial nucleotide biosynthesis *in vitro*, which suggests a metabolic complementarity with milk derived glycans (4, 65).

A second biochemical interface involves the LPO pathway. When H_2_O_2_ and SCN^−^ are available, LPO generates hypothiocyanite that displays broad antimicrobial activity in preclinical systems. Mixing neonatal saliva with breast milk provides purine substrates and XO to produce H_2_O_2_ quickly, thereby activating this pathway at mucosal interfaces *in vitro* (4, 61). These mechanisms support a working model in which milk microbes, milk bioactives, and salivary chemistry jointly shape early oral colonization and the downstream seeding of the upper gastrointestinal tract.

Translationally, this integrated view suggests several directions. First, donor milk processing should aim to preserve key bioactive signals and to maintain safety, with rigorous assessment of microbial and biochemical readouts (33). Second, targeted HMO supplementation can be designed to favor infant adapted taxa identified in human cohorts, with mechanistic grounding in preclinical studies (89, 90). Third, saliva informed stratification may help select candidates for probiotic or synbiotic trials, because salivary purines and microbial profiles vary by feeding status, gestational age, and antibiotic exposure (65, 75). Any application requires stepwise validation that begins in neonatal preclinical models and proceeds to controlled human studies that assess colonization dynamics, immune endpoints, and safety (4, 74).

### 5.6 Coordinated support for microbial colonization

The development of a beneficial gut microbiota likely depends on a coordinated influx of nutrients and signals. In human observational studies and preclinical systems, HMOs selectively enrich Bifidobacterium (and, to a lesser extent in early life, *Lactobacillus*), promoting short-chain fatty acids (SCFA) production that has been linked to epithelial health and reduced inflammation (33). In parallel, measured constituents of neonatal saliva, including nucleotides, nucleosides (e.g., adenosine) and free bases (e.g., uracil), serve as salvage substrates for microbial nucleotide biosynthesis *in vitro* (65). Collectively, these milk–saliva inputs may create a metabolically supportive niche favoring beneficial taxa over pathogens, contributing to early microbial imprinting with long-term associations to immune tolerance, allergy risk reduction, and metabolic homeostasis (35, 90). Rather than acting as isolated fluids, breast milk and neonatal saliva function as an integrated interface linking maternal immunity, infant metabolism, and microbial ecology. Consistent with preclinical evidence, their interaction appears to couple direct antimicrobial effects with deeper layers of immune modulation (e.g., macrophage programming, mucin remodeling), underscoring an evolutionarily coordinated strategy to protect the neonate during a period of heightened vulnerability, as illustrated in **Figure 1**.

## 6 Functional gaps in infant formula and pasteurized donor milk: a barrier to immune equivalence

### 6.1 Loss of enzymatic synergy in milk substitutes

A central enzyme in breast milk–saliva crosstalk is XO, which in preclinical systems catalyzes the formation of hydrogen peroxide from salivary purine substrates and, in turn, fuels LPO-mediated production of OSCN^−^. By contrast, infant formula generally lacks XO and pasteurized donor milk shows heat-inactivated XO activity, reflecting manufacturing and processing steps (91, 92). These substitutes also contain far lower levels of key purine substrates (e.g., hypoxanthine, xanthine), which together would be expected to diminish activation of the XO-LPO-OSCN^−^ pathway. Consequently, the capacity for OSCN^−^ generation at mucosal surfaces is likely reduced in formula- or donor milk-fed infants relative to directly breastfed infants, which may help explain observed differences in susceptibility to respiratory and gastrointestinal infections and in epithelial conditioning reported in observational studies (93).

### 6.2 Thermal inactivation of immunomodulatory proteins

While pasteurization is essential for the microbiological safety of donor milk, comparative analyses indicate that heat processing reduces the integrity and/or activity of several heat-labile immunological constituents, including lactoferrin, lysozyme, sIgA and selected growth factors, relative to fresh maternal milk (94). Pre- and post-pasteurization assessments, together with *in vitro* functional assays, further suggest decreased pathogen inhibition and altered immunomodulatory capacity, and mucins/glycoproteins may likewise be susceptible to heat-induced degradation. Collectively, these changes likely attenuate the extent to which pasteurized donor milk can reproduce the mucosal signaling milieu of fresh maternal milk, although the clinical impact on infant outcomes requires cautious interpretation.

### 6.3 Deficiencies in HMO composition and functional diversity

Another limitation is the restricted HMO repertoire in infant formula. Although selected HMOs such as 2’-fucosyllactose have been synthetically incorporated, current formulations do not replicate the structural diversity, receptor specificity, or microbiota selectivity of the >200 HMOs found in human milk (95, 96). Consistent with this difference, observational studies report that formula-fed infants show distinct early colonization patterns, including higher relative abundances of pro-inflammatory taxa such as Enterobacteriaceae and certain Clostridium spp., compared with breastfed infants (97, 98). While causality cannot be inferred from these data, the findings support an association between limited HMO diversity in formula and microbiota profiles less enriched in beneficial taxa.

### 6.4 Clinical implications and practice considerations

These molecular and biochemical constraints align with epidemiological observations. Across observational studies, formula-fed infants show higher risks/incidences of respiratory and gastrointestinal infections, allergic sensitization, and necrotizing enterocolitis compared with breastfed infants (93, 94). When maternal milk is unavailable, pasteurized donor milk provides a partial immunological substitute, especially for preterm infants; however, it does not reproduce the dynamic, bioresponsive features of fresh maternal milk.

To be clear, infant formula and pasteurized donor milk are indispensable in neonatal care, particularly when direct breastfeeding is medically or socially infeasible, and their value cannot be overstated. At the same time, a persistent immunological gap relative to native breastfeeding underscores the need for further innovation and rigorous evaluation. Future directions may include non-thermal processing methods for donor milk, synthetic-biology approaches to expand HMO diversity, strategies informed by the microbiota that leverage beneficial taxa and microbiota-derived products, and preclinical assessment of strategies to reconstitute XO–LPO activity in formula products. In the meantime, the priority remains to support and enable direct breastfeeding whenever feasible, recognizing it as irreplaceable: it provides optimal nutrition and a co-regulated immunological system shaped by the coevolution of mother and infant, and it also fosters a unique relational context with well-documented psychological and developmental benefits for both (99, 100). A comparative overview of the immunological functionalities of native breast milk, pasteurized donor milk, and infant formula is provided in **Table 2**.

**Table 2:** Comparative immunological functionality of breast milk, pasteurized donor milk, and infant formula.

Immunological feature	Breast milk	Donor milk (pasteurized)	Infant formula
Xanthine oxidase (XO) activity	Present	Inactivated by heat	Absent
Hypoxanthine/Xanthine	Present in saliva	Variable	Absent
Lactoperoxidase activity	High	↓ Reduced	Absent
Immunoglobulins (sIgA)	Functional	↓ Denatured	Not human Ig
Human milk oligosaccharides (HMOs)	Full spectrum (>200 types)	↓ Partial loss	Limited synthetic types
Mucins and glycoproteins	Intact	↓ Heat-damaged	Absent
Growth factors (EGF, TGF-β)	Present	↓ Degraded	Absent
Microbiota support	Optimized	↓ Compromised	Pro-inflammatory microbiota
Supports OSCN^−^ antimicrobial system	Yes	No	No

Data summarized from Al-Shehri et al. (91)Harrison (92)Akinbi et al. (93)Silvestre et al. (94)Triantis et al. (95)Wiciński et al. (96)Duman et al. (97)Inchingolo et al. (98).

## 7 Clinical and translational implications: harnessing breast milk–saliva synergy for neonatal health

The intricate biochemical and immunological interplay between breast milk and neonatal saliva is more than a biological curiosity. It presents a blueprint for translational innovation in neonatal care. As evidence accumulates supporting the functional synergy of this maternal–infant interface, the imperative grows to translate these insights into actionable clinical and pharmaceutical strategies. This section outlines how key elements of this interaction can be repurposed, reengineered, or mimicked to benefit neonates, particularly those deprived of maternal breastfeeding.

### 7.1 Translational framework for milk–saliva synergy

Translating the cooperative biology of breastfeeding into practice requires an integrated pathway that links mechanism, modeling, measurement, and evaluation. Engineered *in vitro* systems can recreate oral and mucosal conditions under controlled shear, pH, redox state, residence time, and microbial context. Synthetic biology and microfluidic platforms allow systematic combination of milk derived bioactives, salivary substrates, and prebiotics, enable high throughput screening, and provide a scalable way to study interindividual variability related to feeding status, gestational age, and antibiotic exposure (101–103). In this framework, *in vitro* engineering serves as the bridge between discovery and downstream testing.

Measurement comes next. Saliva based diagnostics should be developed and standardized to quantify purine metabolites, redox markers, immunoglobulins, and cytokines that reflect early life mucosal readiness and that can be tracked across feeding contexts and clinical states (4, 62, 82). Assay validation should include precision, biological range, and longitudinal stability in diverse neonatal populations, including preterm and immunocompromised infants. Design of nutritional and bioactive interventions should extend beyond caloric adequacy. Priority elements include diversified human milk oligosaccharides and glycan mimetics, stabilized immunoglobulins, lactoferrin mimetics, and enzymatically competent systems that emulate natural milk and saliva chemistry. Formulation science should consider bioencapsulation and protection of bioactivity during storage, gastric passage, and intestinal digestion (33, 95, 96, 104).

Preclinical evaluation should define mechanism, dose response, and the kinetics of oxidant generation and clearance at mucosal surfaces, together with tissue specific safety margins in neonatal models that approximate human physiology. This is essential because hydrogen peroxide and hypothiocyanite participate in host defense and epithelial signaling, which creates potential for benefit as well as risk if exposure exceeds physiological ranges (77, 78). Where possible, engineered *in vitro* platforms should be parameterized with human data and used to prioritize candidates before animal testing. Clinical studies should proceed in stages. Early trials can focus on feasibility, biomarker modulation, and safety in carefully selected populations, followed by efficacy trials that measure infection outcomes, mucosal integrity, and microbiota development. Designs should incorporate stratification by delivery mode, perinatal antibiotic exposure, maternal genotype such as FUT2 secretor status, and feeding modality, since these factors may condition both baseline mucosal ecology and the response to interventions (93).

**Figure 2:**
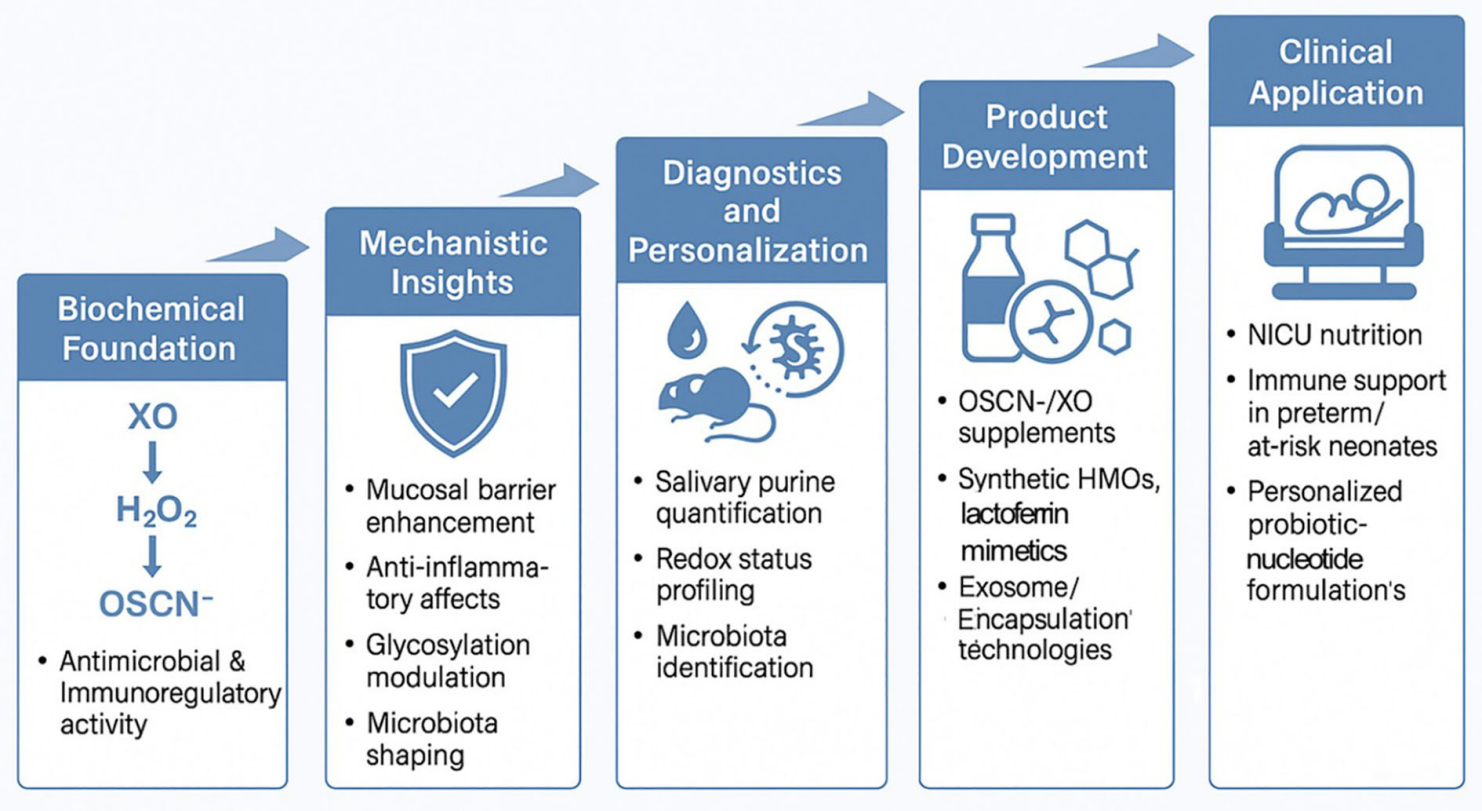
Translational roadmap from breast milk–saliva biochemistry to clinical application in neonatal immune support. This schematic diagram outlines the stepwise translation of the biochemical synergy between breast milk and neonatal saliva into potential clinical applications. The foundational XO–H_2_O_2_–OSCN^−^ cascade exhibits antimicrobial and immunoregulatory activities. Mechanistic insights reveal effects on mucosal barrier function, inflammation, glycosylation, and microbiota shaping. These inform diagnostic and personalization strategies, including salivary purine analysis and redox profiling. Product development avenues include OSCN^−^/XO-based supplements, synthetic HMOs, lactoferrin mimetics, and advanced delivery platforms such as exosomes. Ultimately, these strategies aim to support neonatal intensive care unit (NICU) nutrition, bolster immunity in preterm or at-risk neonates, and enable personalized probiotic–nucleotide formulations.

Together, this framework links mechanistic insight to practical evaluation. It uses engineered models to de-risk candidates, standardized diagnostics to measure relevant biology, and phased preclinical and clinical studies to establish safety and benefit. **Figure 2** summarizes how these components align from biochemical mechanisms to clinical testing.

### 7.2 Limitations and controversies

Evidence for H_2_O_2_ generation by milk XO acting on salivary purines, and for downstream OSCN^−^ formation by LPO, is derived mainly from *in vitro* and other preclinical systems; whether comparable concentrations arise on human mucosal surfaces during feeding remains uncertain (4). Because H_2_O_2_ and OSCN^−^ participate in host defense and epithelial signaling, excessive exposure could disrupt redox homeostasis and provoke inflammation in neonatal tissues. Quantitative dose–response relationships, production and clearance kinetics during feeding, and tissue-specific safety margins have not been defined and require systematic study in relevant neonatal models before clinical consideration (77, 78). Reports of viable or DNA-detectable bacteria in human milk support possible maternal to infant transfer, but low biomass sampling is vulnerable to contamination and requires rigorous controls and strain-resolved methods to distinguish signal from artifact (74, 75, 86). Several attractive mechanisms, including HMO-mediated barrier maturation and pathogen decoy activity, are robust in cell and animal models yet remain incompletely verified in human infants (33, 42, 43). Interindividual variability in neonatal saliva, including wide ranges of hypoxanthine and xanthine, may further modulate the magnitude of milk–saliva reactions and contribute to heterogeneous outcomes (4).

## 8 Conclusion and future perspectives

Breast milk–saliva interactions represent an underappreciated mucosal defense axis that integrates enzymatic catalysis, immunomodulatory cues, and microbiota-shaping factors. Rather than being merely additive, this system appears synergistic, generating antimicrobial effectors, modulating immune-gene expression, and promoting epithelial glycosylation in ways unattainable by either fluid alone. A key translational implication is that infant formulas and pasteurized donor milk, which generally lack the requisite enzymes and substrates for this interaction, may be limited in conferring comparable mucosal protection. The translational potential of breast milk–saliva synergy, from its biochemical foundation to clinical implementation, is illustrated in **Figure 2**. This roadmap outlines how mechanistic insights into mucosal immunity, redox balance, and glycosylation can inform diagnostic development, guide product innovation (such as OSCN^−^-based supplements and synthetic HMO formulations), and ultimately support personalized neonatal care, particularly in NICU settings and vulnerable infants. In summary, translating the cooperative biology of breastfeeding into clinical and nutritional tools may help narrow the immunological gap in neonatal care. These approaches are grounded in evolutionary biology and will require robust preclinical and clinical validation before broad implementation.

Future research should prioritize: (1) elucidating the molecular mechanisms involved in this cross-compartment communication; (2) developing and preclinically evaluating synthetic or recombinant approaches to recapitulate the XO–LPO–OSCN^−^ axis; (3) integrating saliva-based diagnostics to assess mucosal immune readiness; and (4) engineering next-generation nutritional formulations that restore or mimic milk–saliva synergy. Recognizing breast milk and neonatal saliva as a coordinated immune unit, rather than isolated fluids, could help reframe the design of early-life nutritional and immunological interventions.
